# The complex transcriptional landscape of the anucleate human platelet

**DOI:** 10.1186/1471-2164-14-1

**Published:** 2013-01-16

**Authors:** Paul F Bray, Steven E McKenzie, Leonard C Edelstein, Srikanth Nagalla, Kathleen Delgrosso, Adam Ertel, Joan Kupper, Yi Jing, Eric Londin, Phillipe Loher, Huang-Wen Chen, Paolo Fortina, Isidore Rigoutsos

**Affiliations:** 1 Cardeza Foundation for Hematologic Research, Division of Hematology, Department of Medicine, Thomas Jefferson University, Philadelphia, PA, USA; 2 Cancer Genomics Laboratory, Kimmel Cancer Center, Thomas Jefferson University, Philadelphia, PA, USA; 3 Computational Medicine Center, Thomas Jefferson University, Philadelphia, PA, USA

**Keywords:** Platelet, Transcriptome, Ribosomal RNA, Non-coding RNA, miRNA, Repeat elements, Antisense transcripts

## Abstract

**Background:**

Human blood platelets are essential to maintaining normal hemostasis, and platelet dysfunction often causes bleeding or thrombosis. Estimates of genome-wide platelet RNA expression using microarrays have provided insights to the platelet transcriptome but were limited by the number of known transcripts. The goal of this effort was to deep-sequence RNA from leukocyte-depleted platelets to capture the complex profile of all expressed transcripts.

**Results:**

From each of four healthy individuals we generated long RNA (≥40 nucleotides) profiles from total and ribosomal-RNA depleted RNA preparations, as well as short RNA (<40 nucleotides) profiles. Analysis of ~1 billion reads revealed that coding and non-coding platelet transcripts span a very wide dynamic range (≥16 PCR cycles beyond β-actin), a result we validated through qRT-PCR on many dozens of platelet messenger RNAs. Surprisingly, ribosomal-RNA depletion significantly and adversely affected estimates of the relative abundance of transcripts. Of the known protein-coding loci, ~9,500 are present in human platelets. We observed a strong correlation between mRNAs identified by RNA-seq and microarray for well-expressed mRNAs, but RNASeq identified many more transcripts of lower abundance and permitted discovery of novel transcripts.

**Conclusions:**

Our analyses revealed diverse classes of non-coding RNAs, including: pervasive antisense transcripts to protein-coding loci; numerous, previously unreported and abundant microRNAs; retrotransposons; and thousands of novel un-annotated long and short intronic transcripts, an intriguing finding considering the anucleate nature of platelets. The data are available through a local mirror of the UCSC genome browser and can be accessed at: http://cm.jefferson.edu/platelets_2012/.

## Background

Platelets are circulating peripheral blood cells that emerge from the human bone marrow to function as critical components in basic physiological processes such as hemostasis, wound healing, inflammation, angiogenesis and the pathophysiology of tumor metastases. Platelets that exhibit functional extremes convey a commensurate increased risk for bleeding or thrombosis. Notably, the propensity for such extremes has been shown to be heritable [1-3]. Nonetheless, an understanding of the responsible genes and underlying mechanisms remains limited to date. In this regard, genome wide association studies (GWAS) have identified loci associated with platelet number, platelet volume and *ex vivo* platelet aggregation [4,5], but the effect sizes have been quite small. Furthermore, most of the identified loci are not in protein-coding genomic regions. Thus, new approaches are needed to query the repertoire of platelet transcripts.

The platelet transcriptome is a reflection of the megakaryocyte RNA content at the time of (pro)-platelet release, subsequent splicing events, selective packaging and platelet RNA stability, and can provide important insights into platelet biology [6]. Platelets are known to contain messenger RNAs (mRNAs) and indeed most studies support a strong correlation between the platelet’s protein-coding transcriptome and its proteome [7,8]. Platelets also include unspliced pre-mRNAs, rRNAs, tRNAs and microRNAs (miRNAs) [9-11]. Most platelet studies to date have characterized the platelet transcriptome using microarrays and SAGE [12-18]. A recent effort compared human and mouse platelet transcriptomes with the help of deep-sequencing of poly-adenylated, long RNA transcripts [10].

The emerging important roles that non-coding RNAs (ncRNAs) play in a cell [19], their interactions with one another and with protein-coding transcripts [20-25], and the speed by which many categories of ncRNAs [26] burst onto the scene suggests that their involvement in biological processes remains largely unexplored. This is particularly true of platelets where an accurate understanding of the transcriptome has both biological (improved understanding of platelet protein translation and the mechanisms of megakaryocyte/platelet gene expression) and clinical (novel biomarkers of disease) relevance.

Because the content and properties of nuclear and cytoplasmic transcripts vary [27-29], the anucleate human platelet represents a unique model for characterizing post-transcriptional gene expression. In light of the above, we deep-sequenced a) a total RNA preparation, b) a ribosomal-RNA depleted RNA preparation, and c) a short RNA preparation for each of the four individuals. Our results have been embedded in a local mirror of the UCSC genome browser and can be examined interactively at http://cm.jefferson.edu/platelets_2012/.

## Results

We carried out transcriptome sequencing of total RNA isolated from leukocyte-depleted platelet (LDP) preparations from four healthy adults (hereafter referred to as 1N1, 2N2, 3N3, 4N4). LDPs were prepared by density centrifugation of citrated whole blood followed by immunodepletion of CD45+ leukocytes [11]. This preparation yielded fewer than 1 leukocyte per 5 million platelets. For each individual, we constructed three libraries: a) long (≥ 40 nucleotides) total RNA, b) long RNA depleted of rRNA, and c) short (< 40 nucleotides) RNA. All sequencing was carried out on an Applied Biosystems/Life Technologies (AB/LT) SOLiD™ system.

### Read mapping across the genome

The reads from each of the 12 generated datasets were mapped separately on each chromosome and strand of the human genome (assembly hg19) using the BWA program [30] and the protocol described in Methods. The non-uniform coverage of protein-coding transcripts by next generation sequencing reads has been documented before [31] and was encountered in our analysis as well. Table 1 shows the average numbers of obtained and mapped reads for each of the three library types (long total, long rRNA-depleted, and short RNA). Notably, mitochondrial transcripts represented more than half of the uniquely mapped long reads (58.1% long total, 65.1% long rRNA-depleted, 1.3% short), something also encountered by other unbiased methods such as SAGE [12].

**Table 1:** Summary of uniquely mapped reads

**Library**	**Sequenced reads**	**Uniquely mapped reads**
Long, Total RNA	85,526,881	30,465,049 (35.6%)
Long, rRNA-depleted RNA	57,581,167	19,978,474 (34.7%)
Short RNA	104,965,977	32,433,513 (30.9%)

Shown are averages from platelet RNA for each library type and for all subjects.

### Estimating the abundance of protein-coding transcripts in platelets

We devised a scheme (see Methods) for estimating the expression levels of protein-coding transcripts from RNA-seq reads. To estimate transcript abundance, we normalized for transcript length and scaled using the expression levels of the β-actin isoform with ENSEMBL identifier ENST00000331789. This scheme was very effective (see below) and provided us the ability to appropriately scale expression *within* a read-set and to compare expression levels *across* read-sets. This β-actin transcript was quite abundant in platelets, present at approximately 15.0 ± 1.5 cycles of PCR containing the equivalent of 10 ng of total RNA, and shows the least amount of variation (± ~3%) across the analyzed samples (Additional file 1: Table S1). Pairwise comparisons (Pearson correlation) of our mRNA data after normalizing with *GAPDH* and two additional stable platelet transcripts, *PPBP* and *B2M*, revealed data virtually identical to those originally obtained using *ACTB*. Notably, the isoforms of the housekeeping gene GAPDH, which is often used as an expression normalizer, exhibited a substantial expression variation upon rRNA depletion (-70% to +130% depending on the specific GAPDH transcript that was considered). It will be important for future platelet RNAseq studies with larger numbers of subjects to confirm these observations pertaining to the isoforms of these two commonly utilized platelet “normalizers.”

**Figure 1:**
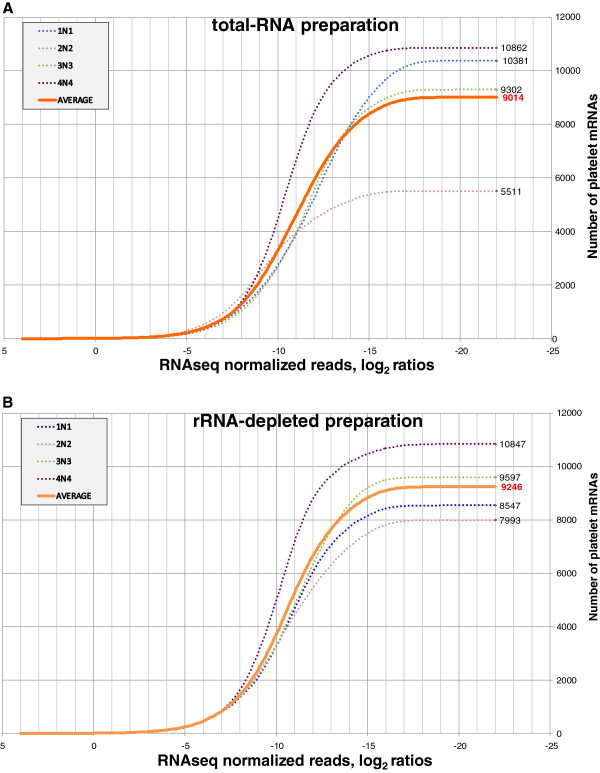
**Estimates of platelet expressed mRNAs. A**) Total RNA. **B**) rRNA-depleted RNA. The x-axis shows the RNA-seq read number in log2 ratios normalized to β-actin; the y-axis shows the number of expressed mRNAs. The result for total RNA from donor 2 N2 is an outlier with lower abundances.

We used the most abundant isoform among those derived from an individual protein-coding gene to represent the gene. Figure 1 shows the number of protein-coding genes as a function of the level of normalized expression. This approach revealed different estimates of protein-coding genes that are present at a given level of abundance between total and rRNA-depleted RNA preparations. The finding underscores that estimates of expressed genes were more similar amongst different subjects for high abundance genes (leftwards in Figure 1), and that there was substantial inter-individual variation in total transcript estimates when considering the less abundant genes (rightwards in Figure 1). Protein-coding transcripts for each of the four samples whose expression was supported by the RNA-seq data are shown in Additional file 2: Table S2A (total RNA) and Additional file 3: Table S2B (rRNA-depleted RNA). It is worth stressing that our normalization scheme enabled us to compare expression levels across all preparations.

### RNA-seq vs. qRT-PCR

**Figure 2:**
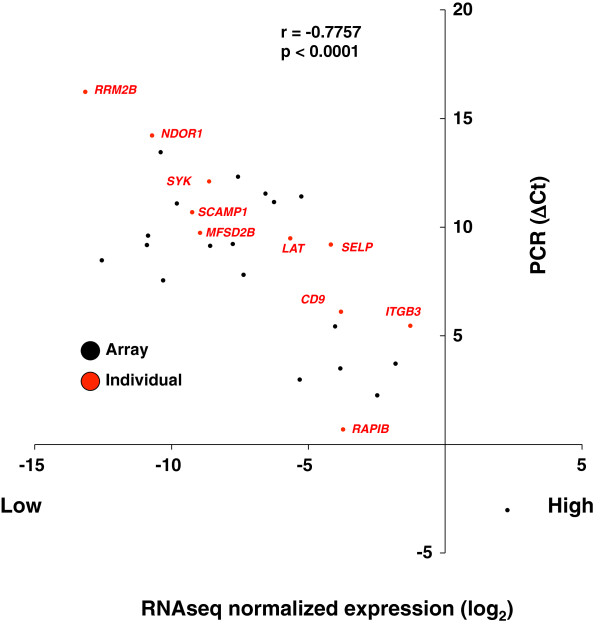
**Correlation of platelet mRNA levels assessed by RNA-seq and qRT-PCR.** ΔC_t_ values obtained by qRT-PCR (y-axis) were plotted against the log_2_-normalized transcript determined by RNA-seq (x-axis). Both methods normalized to β-actin expression. Transcripts were considered “present” in the qRT-PCR with a cycle threshold of ≤35. RNA-seq transcripts were considered that were no lower than a normalized log2 expression value of -15 (i.e., 15 PCR cycles [≥ 1/32,768^th^] of β-actin expression). The transcript in the right lower quadrant represents β_2_-microblobulin, which is expressed at a higher level than β-actin. Black points derive from microarray; red points were selected as known, representative platelet genes.

We sought to determine the correlation between our RNA-seq normalization approach and qRT-PCR on the same RNA samples. We queried 2 collections of genes: 1) 10 transcripts that exhibited a broad range (> 3 orders of magnitude, i.e. more than 10 PCR cycles) of normalized read counts, 6 of which are well-studied in platelets; and, 2) 89 transcripts for GPCR signaling proteins from a commercial platform, 19 of which were detected using both RNA-seq and qRT-PCR. Figure 2 shows a very high correlation (Pearson r-value of 0.7757 at a p-value <0.0001) for transcripts detected by both methodologies, indicating that our approach of estimating transcript abundance from RNA-seq data is accurate over a wide range of transcript expression levels.

### RNA-seq vs. microarrays

**Figure 3:**
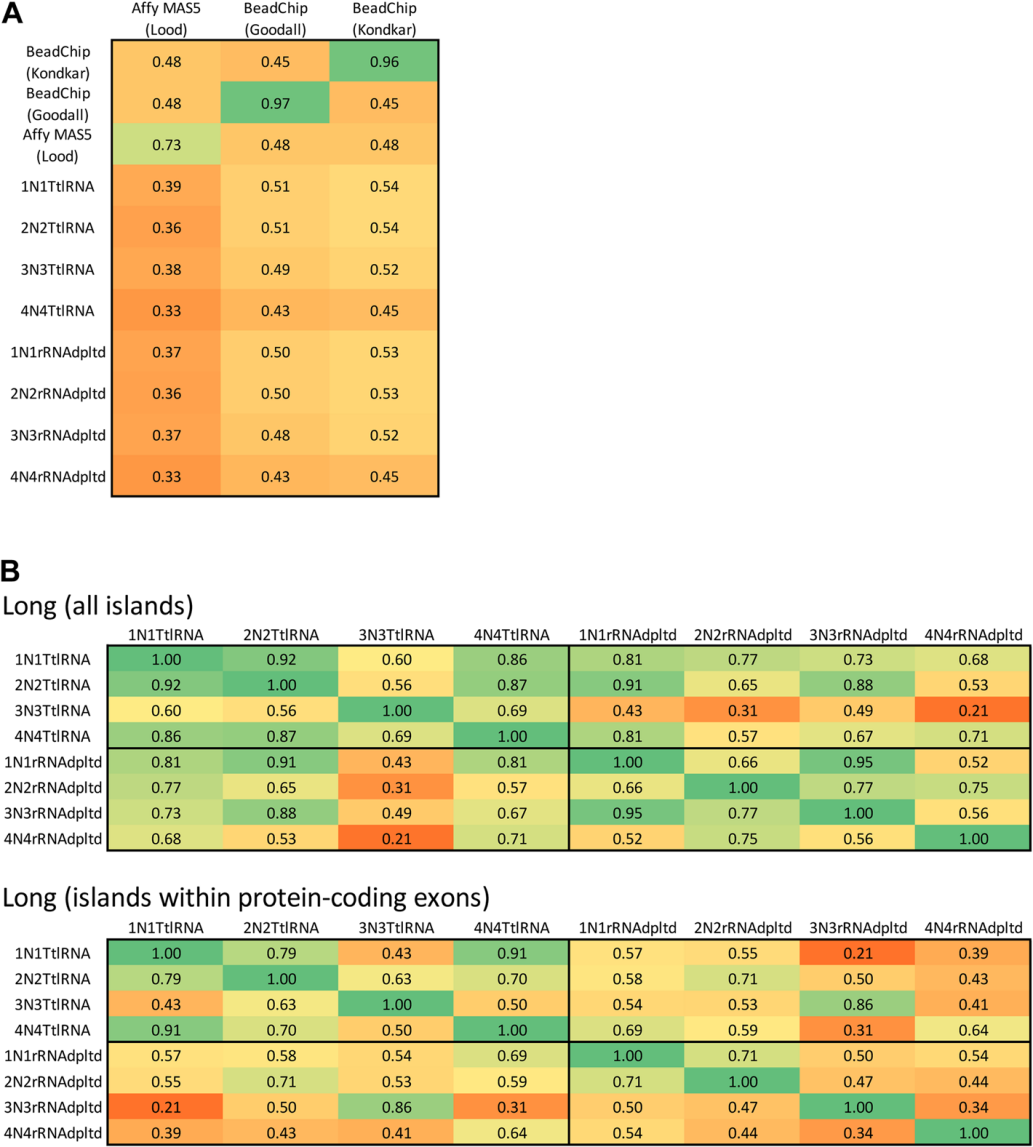
**Correlation heatmap matrix for RNA-seq vs. microarray analysis of the platelet transcriptome. A**) To compare the protein-coding transcripts as deduced from RNA-seq and previous microarray analyses (Affymetrix GeneChip and Illumina BeadChip) and also microarrays with one another, we used a Spearman correlation computed from the union of protein-encoding genes (13,691 in all) that were represented on at least one of the platforms. **B**) To compare the RNA-seq datasets with one another, we computed Pearson’s correlation between the genomic transcript profiles obtained by each dataset. In both **A**) and **B**), each square lists the correlation coefficient value between the corresponding profiles; also, the color-coding convention is the same in order to facilitate comparisons.

We also compared protein-coding transcripts from RNA-seq data with previously published microarray datasets of the platelet protein-coding transcriptome [15,17,32]. The three microarray datasets exhibited reduced pair-wise correlation with one another, perhaps the result of a dependence on the used platform and differences in the sample sources and preparations (Figure 3A). In contrast, there is a high and significant pair-wise correlation among the RNA-seq datasets (Figure 3B). In light of these observations, it is not surprising that there was less correlation between any RNA-seq and any microarray set (Figure 3A).

### Adverse impact of ribosomal-RNA depletion on the estimates of mRNA abundance

Having established the appropriateness of our normalization scheme, we sought to determine the potential impact of the depletion of ribosomal RNAs on the estimate of relative abundance of the various protein-coding transcripts. To this end, we computed the ratios of the normalized abundance of transcripts between the total and the rRNA-depleted RNA preparations. In an effort to be conservative, and based on the data in Figures 1 and 2, we only considered protein-coding transcripts with an estimated abundance that was ≥ 2^-10^ times that of β-actin, and kept only those whose absolute ratio value was ≥ 2× between the two preparations. Unexpectedly, the number of affected protein-coding genes was high, ranging from 745 (sample 4N4) to 2,341 (sample 2N2) genes (Additional file 4: Table S3). Considering the stringency of our criteria, the true number of affected protein-coding transcripts is very likely higher. These findings suggest that the ribosomal RNA depletion step adversely and extensively impacts the relative abundance of protein-coding transcripts within a sample and, by extension, the accurate estimate of the transcripts’ expression levels. The situation is further aggravated by the fact that the magnitude of this impact appears to be transcript-dependent and thus non-uniform: as can be seen from Additional file 4: Table S3, the ratio of the normalized expression between the total and rRNA-depleted preparations spans a wide spectrum of values in all four samples. Of particular note are the members of the RNA interference pathway *DGCR8, DROSHA, XPO5, DICER1, EIF2C1, EIF2C2, EIF2C3*, and *EIF2C4* (Table 2): all of them exhibited large differences (up to 32-fold) between the total and rRNA-depleted preparations.

**Table 2:** rRNA depletion alters the relative quantities of transcripts

**Gene**	**Range of estimate ratios (log-scale)**
*DICER1*	−1.35 to 2.23
*DROSHA*	−0.97 to 1.88
*EIF2C1*	−0.18 to 2.10
*EIF2C2*	−0.30 to 1.23
*EIF2C3*	−0.72 to 4.06
*EIF2C4*	−1.19 to 0.37
*XPO5*	−2.50 to 2.09

The range (in log2-units) of the observed ratio “normalized-gene-abundance-in-total over normalized-gene-abundance-in-depleted” from four different biological samples and for genes relevant for the RNA interference pathway. Ratios were derived from pairs of total and depleted preparations generated from the same biological sample.

### Enriched categories of platelet protein-coding transcripts

**Figure 4:**
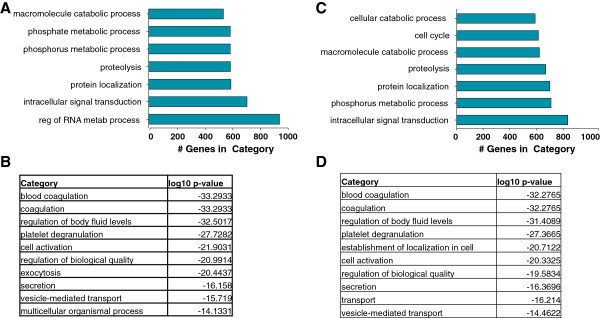
**Gene Ontology (GO) analysis of the platelet transcriptome by RNA-seq.** Top-ranking biological processes by gene number (panels **A** and **C**) and by categories (panels **B** and **D**) that emerge from a GORILLA analysis of those protein-coding transcripts common to the four sequenced individuals. GO terms and p-values were computed and are shown separately for the total RNA (panels **A** and **B**) and the rRNA-depleted preparations (panels **C** and **D**). Note that the GO category of "coagulation" includes all aspects of platelet biology. See also Figure S1.

We generated the intersection of the expressed protein-coding transcripts across all four samples and ranked them according to abundance. We processed separately the total RNA and the rRNA-depleted preparation. Using GORILLA [33] with the four ranked lists corresponding to each of the two preparations we calculated GO term enrichments with an eye towards assessing whether the platelet protein-coding transcriptome was enriched for certain biological characteristics. Figure 4 shows the top-ranking functional annotation clusters for the biological process category, the number of genes sharing each term, and the associated p-value (log10). As expected, biological process terms such as coagulation, platelet degranulation, etc. were over-represented in the platelet transcriptome and both preparations. Additional analyses of GO terms pertaining to cellular compartment and molecular function are shown in Additional file 1: Figure S1A-H.

### Platelet miRNAs

We also queried the presence of miRNAs in the platelet transcriptome. Additional file 5: Table S4 shows the complete set of miRNAs whose expression was supported by the RNA-seq data for each of the four samples. The expression data were normalized with the help of *SNORD44*; *SNORD44* was selected because of its abundance and observed general stability across very diverse tissues. The table reveals that the expression for hundreds of miRNAs was ≥ 32 times higher than *SNORD44*, suggesting that the platelet transcriptome is rich in miRNAs, a finding also reported by Landry et al. [34]. Unique to our analysis is that we distinguish between the two potential products of a miRNA precursor, namely 5p and 3p, and examine each product’s expression separately (Additional file 1: Figure S2 explains why this is important).

### Pseudogenes

In light of recent work highlighting the importance of pseudogenes in regulating miRNA-mediated repression of targeted mRNAs [20,21], we analyzed our sequenced read sets for evidence of pseudogene transcription. To this end, we used the pseudogene definitions contained in Release 63 of the ENSEMBL database: this Release lists 11,983 transcripts corresponding to 11,158 genes. We found pseudogene loci to be highly enriched across all four samples and in both the total and rRNA depleted preparations (see Additional file 1: Table S5 for details). Notably, the observed enrichment values mirrored one another across the preparations.

### Repeat elements

We also focused on the repeat element category of characterized transcripts. In particular, we computed enrichment values for both sense and antisense transcripts for each of the 116 families of elements that are recognized by RepeatMasker [35] and separately for each of the four samples and the three preparations (total and rRNA-depleted long RNA, short RNA) – a total of 12 sets. Additional file 1: Table S6 and Additional file 1: Table S7 show that several repeat family loci give rise to both long and short platelet RNA transcripts.

### Other categories of non-coding RNAs

Recently, a novel class of long ncRNAs, the “long-intergenic non-coding RNAs,” or lincRNAs for short, has received a lot of attention [23,36]. LincRNAs number over a thousand members, yet with the exception of a handful of reports [37-39] they remain essentially uncharacterized. Our analysis of the sequenced reads did not reveal any enrichment of the corresponding genomic loci.

### Novel and uncharacterized intronic transcripts

Our work uncovered extensive evidence for the existence of transcripts that originate in the introns of known protein-coding genes. This is of particular significance considering that platelets lack a nucleus. For such an analysis it is imperative to distinguish *bona fide* intronic regions from well-characterized transcripts that are known to be co-located with the introns of protein coding genes. We thus worked with unspliced messenger RNA sequences after first having 'subtracted' all sense instances of the following categories of transcripts: protein-coding and non-protein-coding exons; all known repeat elements; rRNAs; snoRNAs; miRNAs; and, lincRNAs. To this end we used the annotations in Release 63 (June 30, 2011) of the ENSEMBL database. We analyzed each of the four samples and three preparations (total and rRNA-depleted long RNA, short RNA) separately. For the long RNA read sets, we considered intronic real estate if and only if platelet reads covered a minimum of 100 consecutive nucleotides and the covered region had an estimated abundance relatively to *ACTB* of 1:1024 (which is equivalent to a conservative dynamic range of not more than 10 PCR cycles beyond *ACTB*). For the short RNA read sets, we only considered platelet reads mapping to intronic real estate if they were at least 30 nucleotides long and had an estimated abundance relatively to *SNORD44* of 1:64 (which is equivalent to a conservative dynamic range of not more than 6 PCR cycles beyond *SNORD44*). Given the high stringencies of length and abundance, we accepted such a region if at least one of the sequenced samples showed evidence for it. Across the four samples and two long RNA preparations, we found a total of 6,992 *bona fide* intronic regions that give rise to currently uncharacterized long RNA transcripts satisfying the above constraints. We also found an additional 1,236 *bona fide* intronic regions that give rise to currently uncharacterized short RNA transcripts satisfying the above constraints. Notably, these two collections of intronic regions had only 18 members in common suggesting that the two novel populations of (long and short) uncharacterized *bona fide* intronic transcripts originate from distinct genomic loci. Additional file 6: Table S8 lists the genomic coordinates for these two groups of intronic regions.

### Novel and pervasive antisense transcripts

Our analysis also revealed the presence of a substantial number of long and short platelet transcripts that were antisense to known miRNAs, known protein-coding exons, and notably, to known repeat element families. For the miRNA analysis, we processed separately the four read sets from the short RNA preparation. For the protein-coding transcript analysis, we processed separately the eight read sets from the total and rRNA-depleted preparations. For the repeat element analysis, we processed all read sets separately for each of the four sequenced individuals. The following are the 10 miRNA precursors with previously unreported antisense transcripts: *hsa-miR-33b*, *hsa-miR-101*, *hsa-miR-191*, *hsa-miR-219-2*, *hsa-miR-374b*, *hsa-miR-486*, *hsa-miR-625*, *hsa-miR-766*, *hsa-miR-3135b*, and *hsa-miR-4433*. The short platelet RNAs we observed had lengths typical of a miRNA and were transcribed from the strand opposite of that of the known miRNA precursor. Each of the loci listed above generated one or two distinct antisense transcripts, presumably a mature miRNA and its “star” miRNA. There was also a high prevalence of transcripts that were antisense to known protein-coding regions of the genome. Table 3 shows the enrichment in such antisense transcripts that overlap the 5^′^UTRs, 3^′^UTRs or full-length exonic space of known protein-coding transcripts. Enrichment values are notable, independently of whether we computed them in terms of span (which ignores the number of reads sequenced from a genetic locus) or in terms of support (which takes into account the number of reads sequenced from a genetic locus). Unexpectedly, our analyses revealed notable enrichment in both long and short platelet RNAs that were antisense to several known repeat families. Table 4 shows these enrichments for the sequenced short platelet RNA-omes. Additional file 1: Table S9 shows the corresponding values for the long platelet RNA-omes and separately for the total and rRNA-depleted preparations.

**Table 3:** Long platelet RNAs antisense to protein-coding sequences

	**Total RNA (span enrichment)**				**rRNA depleted (span enrichment)**			
**region**	**sample 1**	**sample 2**	**sample 3**	**sample 4**	**sample 1**	**sample 2**	**sample 3**	**sample 4**
5^′^UTR	28.28	39.21	34.20	13.53	33.33	40.21	33.32	12.59
3^′^UTR	53.87	64.88	52.59	23.54	64.18	62.58	55.78	21.51
Exons	52.65	69.80	59.73	24.01	56.53	66.36	59.95	21.46
	**Total RNA (support enrichment)**				**rRNA depleted (support enrichment)**			
**region**	**sample 1**	**sample 2**	**region**	**sample 1**	**sample 2**	**region**	**sample 1**	**sample 2**
5^′^UTR	2.89	4.22	6.29	3.79	6.02	4.32	11.94	9.35
3^′^UTR	7.77	8.87	8.86	7.24	17.61	7.41	21.97	12.57
Exons	11.09	11.32	12.06	8.61	14.88	8.84	25.31	13.20

Enrichment values are shown for the four samples and for sequenced long transcripts that are antisense to the 5^′^UTRs, 3^′^UTRs or full-length exons of known protein-coding transcripts. For comparison purposes, we report span enrichment and support enrichment values, separately for the total and rRNA-depleted preparations. Note the consistency of the enrichment across the four subjects and the two preparations.

**Table 4:** Short platelet RNAs antisense to repeat elements

	**Repeat family**	**Span enrichment**		**Repeat family**	**Span enrichment**
**sample 1 / Short**	DNA?.DNA?	2.08	**sample 2 / Short**	LTR?.LTR?	2.88
	SINE.SINE	2.05		tRNA.tRNA	2.38
	LINE.Dong-R4	1.85		DNA?.DNA?	2.00
	SINE?.SINE?	1.82		LINE.Dong-R4	1.95
	LTR.ERVL?	1.80		snRNA.snRNA	1.76
	tRNA.tRNA	1.79		SINE?.SINE?	1.75
	LINE.RTE-BovB	1.73		Satellite.acro	1.70
	SINE.tRNA	1.64		SINE.SINE	1.64
	rRNA.rRNA	1.62		Unknown.Unknown	1.63
	snRNA.snRNA	1.62		scRNA.scRNA	1.62
	DNA.hAT-Blackjack	1.60		LTR.ERVL?	1.59
	DNA.TcMar-Mariner	1.60		SINE.tRNA	1.57
				LTR.LTR	1.55
				DNA.hAT-Blackjack	1.53
					
**sample 3 / Short**	DNA.Merlin	2.68	**sample 4 / Short**	LINE.L1?	2.72
	Unknown?.Unknown?	2.35		tRNA.tRNA	2.49
	LTR?.LTR?	2.14		DNA.Merlin	2.40
	LINE?.Penelope?	2.02		SINE?.SINE?	2.09
	LINE.Dong-R4	1.95		LINE.RTE-BovB	2.02
	tRNA.tRNA	1.83		SINE.tRNA	1.96
	scRNA.scRNA	1.78		LTR?.LTR?	1.88
	SINE.SINE	1.74		rRNA.rRNA	1.87
	LTR.ERVL?	1.72		DNA?.DNA?	1.79
	SINE.tRNA	1.69		Unknown.Unknown	1.71
	DNA?.DNA?	1.66		LINE.Dong-R4	1.69
	rRNA.rRNA	1.65		SINE.SINE	1.64
	DNA.PiggyBac?	1.65		Unknown?.Unknown?	1.56
	snRNA.snRNA	1.62		snRNA.snRNA	1.55
	SINE.Deu	1.60		DNA.hAT-Blackjack	1.54
	LTR.LTR	1.59		LTR.LTR	1.54
	Unknown.Unknown	1.58			
	DNA.hAT-Blackjack	1.55			
	LINE.RTE-BovB	1.54			
	DNA.TcMar-Mariner	1.52			

Enrichments values are shown for the four samples and for sequenced short transcripts that are antisense to known categories of repeat elements. Note the prevalence of antisense transcripts to tRNAs, LINEs, SINEs and DNA transposons. All values represent span enrichments. The use of a question mark (“?”) next to a repeat family category is inherited from RepeatMasker’s notation convention and indicates that the corresponding sequences are likely members of the stated repeat family as per RepeatMasker’s analysis.

### 'Orphan' reads

We use the characterization ‘orphan’ to refer to those RNA-seq reads that could not be mapped on the human genome using our default parameter settings. To ensure that we exhausted the possibilities, and in an effort to address the potential identities of unmapped transcripts, we conducted additional read mapping with alternative computational settings and using curated datasets.

First, and in light of recent reports of extensive editing of RNA transcripts [40] we used the BWA algorithm [30] with higher-than-default sensitivity settings: in particular, we permitted up to six mismatches in the context of BWA’s length-dependent scheme for allowing mismatches. We used this lenient parameter setting for both the total and rRNA-depleted preparations (8 sets of reads in total). In each case, we were able to map an additional approximately four million reads (~6.5% of the original set of sequenced reads). Additional file 1: Table S10 provides relevant detailed statistics.

Since we used the full genome’s sequence to map the sequenced reads the formal possibility remains that perhaps a significant portion of the orphan reads originate from the exon-exon junctions of spliced protein-coding transcripts. Thus, our subsequent investigation used the 598,379 exons listed in Release 63 of ENSEMBL to combinatorially enumerate all possible exon-exon junctions using the known, non-overlapping exons of all 51,055 protein-coding and non-protein-coding genes contained in the Release. This gave rise to 12,382,819 junctions on which we attempted to map the orphan reads. Across all read sets that were sequenced from the total RNA preparations, an average of 185,026 reads were mapped onto the exon-exon junction set. The corresponding number for the sets obtained from the rRNA-depleted preparations was 191,736 reads. In both cases, only a very small fraction of the reads mapped to exon-exon junctions.

Lastly, we examined the possibility that the orphan reads originate from the highly polymorphic human leukocyte antigen (HLA) region of chromosome 6. To this end, we used the 6,944 sequences contained in Release 3.5 of the IMGT/HLA database [41,42] and searched them with BWA and standard settings. An average of 5,601 (total RNA) and 5,564 (rRNA-depleted RNA) reads were mapped to this region suggesting that transcripts from the HLA regions do not contribute in any significant manner to the platelet transcriptome.

### Data Access

Results have been embedded in a local mirror of the UCSC genome browser and can be examined interactively at http://cm.jefferson.edu/platelets_2012/.

The data set supporting the results of this article is available in the NCBI/GEO repository, accession number SRA062032, http://www.ncbi.nlm.nih.gov/sra. The data sets supporting the results of this article are included within the article and its additional files.

## Discussion

### The cellular transcriptome

A prominent lesson that has emerged from the 1000 Genomes Project is the greater genetic variation in the population than previously appreciated. Transcriptomics is rapidly assuming a more prominent role in the understanding of basic molecular mechanisms accounting for variation within the normal population and inherited disease. We have sequenced RNA from the leukocyte-depleted platelets of four healthy individuals and report our findings from the analysis of the long and short RNA transcript populations. In the case of long RNAs, we carried out sequencing of both total and rRNA-depleted RNA. The generated data, accompanying genome browser, and data repository detail the totality of RNA species present in the anucleate human platelet. We are unaware of prior efforts that have provided as comprehensive a transcriptome evaluation of any human cell as offered in our report. Our approach serves as a roadmap for future transcriptome analyses and the findings have important implications for the understanding of the transcriptome and the role of platelets in health and disease.

We utilized a distinct approach to the elucidation of the platelet transcriptome that, as we discovered, exhibits an extraordinary complexity. Features of our approach include: 1) the use of the anucleate platelet that decouples the nuclear and cytoplasmic transcriptomes; 2) the use of total RNA and not poly-A enriched RNA; 3) the use of a next generation sequencing platform (AB/LT SOLiD) that generated the high enough read numbers needed to provide the required resolution power; 4) the explicit evaluation of the impact of the ribosomal RNA depletion step prior to sequencing; 5) an enhanced mapping protocol that ensured exhaustive mapping of the sequenced reads on the un-masked human genome and the exclusion of reads that could not be mapped uniquely; and, 6) the explicit search for the presence or absence of RNA species that either have not been previously discussed in the context of platelet biology or that are not currently annotated in the public databases. Findings from our analyses reveal a much more diverse platelet transcriptome than previously appreciated, and include pseudogenes, repeat elements, *bona fide* intronic transcripts, novel short and long RNAs, transcripts antisense to exons and antisense to miRNAs. Our data are publicly available and can be explored interactively through our local mirror of the UCSC genome browser at http://cm.jefferson.edu/platelets_2012/.

### The platelet context

Blood platelets originate from bone marrow precursor megakaryocytes. As such, most platelet RNA results from the transcription of nuclear DNA in the megakaryocyte, and thus reflects the status of the megakaryocyte at the time of platelet release into the circulation. Notably, megakaryocytes from human bone marrow are neither routinely nor easily accessible for biological studies. Megakaryocyte gene transcription responds to numerous normal physiologic and pathologic stimuli. Additionally, anucleate platelets are known to engage in both post-transcriptional processing of RNA and translation of mRNA into protein, in response to external factors [9,43]. Consequently, the platelet transcriptome represents a critical proxy biomarker of both megakaryocyte activity and of the hemostatic, thrombotic, and inflammatory challenges to the organism. These properties in conjunction with the rapidly emerging appreciation of the role of non-coding RNAs in post-transcriptional processing and translation make an inventory of the platelet RNA-ome both timely and important.

Compared to other RNA-evaluating technologies, the current limitations of RNA-seq in general and as applied to platelets are the expense and the need for sophisticated computational analyses that have not yet been standardized or made widely available. As experience with the method progresses and prices drop, these limitations will be offset by the advantages of superior dynamic range, the discovery of novel transcripts, and the simultaneous assessment of expression levels, sequence variants and splice variants, none of which can be achieved using conventional probe-based transcript analysis. A direct digital detection technology (referred to as “Nanostring”) [44] offers the advantage of requiring less starting material, which can be limiting in platelet RNA studies, but this technology is only available for profiling *known* miRNAs or limited sets of *known* mRNAs. Of course, any RNA transcriptome analysis must be considered in the context of potential differences with megakaryocytes. Recently, platelet RNA-seq successfully revealed abnormal splicing events in 1) *NBEAL2*, thus identifying the gene responsible for the Gray Platelet Syndrome [45], and, 2) the RNA-binding protein *RBM8A*, thus uncovering the gene responsible for the TAR (=thrombocytopenia and absent radii) syndrome [46]. Our data will serve as an early and comprehensive reference and resource for other investigators wishing to understand better the normal platelet transcriptome when searching for disease-producing transcript variants. Furthermore, it will serve as a much needed “parts list” of platelet RNAs in the context of studies of RNA-RNA and RNA-protein regulatory interactions. The absence of active transcription makes the platelet an attractive cell type for elucidating and deciphering such higher order regulatory couplings.

RNA-seq is highly sensitive and capable of detecting variability between samples caused by biological differences, technical variation, or environmental influence during sample handling. The samples in our study were processed using a methodology with excellent reproducibility [47] that minimizes technical and environmental factors, and that was able to discover novel genetic and transcriptomic variants regulating platelet biologic function [11,48,49]. However, additional platelet RNA-seq data and analyses from a larger number of subjects is needed to assess the relative contribution of biological versus technical factors contributing to the observed transcript variation.

It is difficult to compare and contrast our study with that of Rowley et al. [10] because of key differences in design, and in the technical and analytic approach. A particular value of the Rowley study is the comparison of human and mouse platelet transcriptomes, which noted some unexpected differences. However, Rowley et al. did a single sequencing run on a pool of 2 human donors, whereas we separately sequenced and provide profiles of long total RNA, long rRNA-depleted RNA, and, short RNA from 4 subjects. The larger number of samples, an increased sequencing granularity, a normalization scheme that allows comparisons and assessment of inter-individual variation, and a wide-ranging analysis of the culled RNA-omes (both protein-coding and non-coding) represent key elements of our work. Additionally, our use of the industry-standard UCSC genome browser for visualizing our data will enable faster access and dissemination of our results.

### The findings

#### Validity of the approach

Comparison of our data to microarray results, both ours and those in the public databases, showed RNA-seq to have significant correlation with microarray for the subset of abundant protein-coding RNAs. GO analyses indicated that the expressed mRNAs were enriched in terms such as coagulation, platelet degranulation, secretion, cytoskeletal dynamics, receptor binding and G-protein signaling. These analyses validate and support RNA-seq and our analytic approach as appropriate for assessing the platelet transcriptome.

#### The number of protein coding transcripts

In this work we confirm and, more importantly, extend earlier platelet transcriptome studies by us and others [10-12,15] in unanticipated ways. Prior platelet work estimated the number of protein-coding transcripts to between 1,500 and 9,000. These earlier efforts neither emphasized nor appreciated the notion that such a count is somewhat of a “moving target.” Our analyses of the RNA-seq data clearly demonstrate that such an estimate and the ability to do cross-sample comparisons depend upon 1) the resolution ability of the used sequencing platform, 2) the read mapping criteria (e.g., use of uniquely mapping reads), and 3) the used “read count” threshold. Within 16 PCR cycles of β-actin, we find ~9,000 mRNAs in the platelets of 4 healthy donors. Relaxed or more stringent criteria provide higher or lower estimates, respectively (Figure 1).

#### Ribosomal RNA depletion

Depletion of ribosomal RNA is considered a standard approach in RNA-seq studies of nucleated cells. Driving the choice is the observation that rRNA makes up ~75-80% of the total amount of cellular RNA. To the best of our knowledge, the impact of rRNA depletion has not been previously studied, certainly not in the context of platelet transcriptome analyses. Importantly, we found that rRNA depletion strongly and adversely impacts the characterization of platelet protein coding transcripts. Indeed, rRNA depletion resulted in variations in abundance estimates that confounded meaningful analyses across samples. Neither we nor others in the field have ascertained the underlying mechanism by which rRNA depletion alters relative mRNA abundances. Our finding does not appear to be a non-specific artifact: not only is the dynamic range of the observed impact very significant but the number of the affected mRNAs is large. In previous work, several authors noted that platelets dock mRNAs to ribosomes and that this process can be selective for features of specific mRNAs [50,51], so it is conceivable that the observed impact of rRNA depletion on mRNA abundance is a platelet-specific event.

#### Novel antisense transcripts

Our analyses unexpectedly revealed the existence of numerous transcripts that are antisense to previously annotated genomic regions. In particular, we discovered consistent enrichment in RNAs that are antisense to the exons of known protein-coding loci across the four healthy donors. We also found enrichment in long RNAs that are antisense to known repeat families. Notably, we found even more pronounced enrichment in *short* RNAs that are antisense to many different repeat families. Naturally occurring antisense transcripts are important regulators of gene expression via interference with translation, RNA masking, etc. [52], and our results suggest the possibility of important, previously unappreciated roles of antisense transcripts in platelet biology.

#### Many more miRNAs

We also discovered a larger number of platelet miRNAs than previously reported, and separately characterized and reported on the two potential products that can be transcribed from a microRNA precursor. Importantly, for 10 miRNA loci, we found and report evidence of transcription of short, miRNA-like-in-length RNAs that are antisense to the known miRNA or its “star” species. Not only are these miRNA antisense species not currently contained in miRBase, but to the best of our knowledge, they have not been reported previously in the platelet context.

#### Intronic transcripts

An additional intriguing finding pertains to our identification of both short and long RNA transcripts that originate from thousands of *intronic* DNA genomic regions and are not currently annotated in the public repositories as known non-protein-coding transcripts from protein-coding loci, miRNAs, ribosomal RNAs, tRNAs, repeat elements, etc. In particular, the intronic loci that give rise to the sequenced *long* platelet RNAs are distinct from the intronic loci that give rise to the sequenced *short* platelet RNAs. It is important to stress that we required a minimum span of 100 nucleotides for those intronic regions that gave rise to the un-annotated long RNAs and a minimum span of 30 nucleotides for those intronic regions that gave rise to the un-annotated short RNAs. Such strict criteria suggest that the actual sources of un-annotated intronic platelet transcripts are more numerous. Although it is possible that intergenic RNAs or transcripts with retained introns are not functional in platelets, there is accumulating evidence that intronic regions likely play rather involved and functionally significant roles in a cell [53-58]. These reports, in conjunction with the data that we have generated, and together with other accounts whereby specific intronic transcripts have been associated with some diseases [45,46] suggest that our resource will be useful reference material for platelet disorders.

#### Transcripts from repeat elements and pseudogenes

One of the unexpected findings that emerged from our work and analyses is the pervasive presence of long and short RNAs that are both sense and antisense to the genomic locations of many families of repeat elements, and nearly all known pseudogenes. The presence of expressed repeat elements *per se* is not new and has been reported previously, e.g. [59-61]. Additionally, many reports have already provided evidence of significant connections between repeat elements and cellular processes in health and disease [62-66]. However, there are several novel and interesting elements that emerged from our analyses and warranted reporting. First, we observed that there were specific categories of repeat elements that were present in our profiles. Second, the present categories seemed to have consistent enrichments across the sequenced individuals. Lastly, the consistency in the profiles among individuals - despite the absence of transcriptional activity - fuels the hypothesis that these repeats are of potential functional significance in the platelet context. However, the considerable numbers of repeat elements and the relative diversity of their categories make it difficult to conjecture what their roles may be. Indeed there is a large number of possibilities that include: the possible formation of endogenous siRNAs or small non-coding RNAs as previously described [67,68]; the possible production of currently unsuspected miRNAs [69,70]; the creation of substrates for miRNA targeting that could then acts as “decoys,” i.e. as competing endogenous RNAs (ceRNAs) that regulate mRNAs [20-22,71,72]; their involvement in previously unrecognized regulatory mechanisms [73-75]; etc. It is also important to note that the observed repeat expression in platelets appears to be ‘marshalled’ and unlike the aberrant expression that has been reported in human cancers [66].

## Conclusion

Our work has revealed a highly complex transcriptional landscape for the anucleate human platelet. The richness and diversity of the present RNA molecules suggests a context where platelet biology transcends protein- and miRNA-centric descriptions. By making available our findings we aim to facilitate the elucidation of previously unappreciated molecular species and molecular interactions. This will eventually permit an improved understanding of the molecular mechanisms that regulate platelet physiology and contribute to serious disorders of thrombosis, hemostasis, and inflammation.

## Methods

### RNA preparation and RNA-seq

The study was approved by the Institutional Review Board of Thomas Jefferson University, and informed consent was obtained from all participants. Highly purified, leukocyte-depleted platelet (LDP) preparations were obtained as previously described [11]. RNA extraction was performed with TRIzol^®^ (Invitrogen, Carlsbad, CA) and RNA quality was assessed by the Agilent bio-analyzer PICO chip. In some experiments, 2 μg of total RNA was depleted of large 18S and 28S rRNA as well as 5S and 5.8S using the RiboMinus Eukaryotic Kit (Invitrogen), which uses biotinylated probes designed against these rRNAs (following the manufacturer‘s protocol). Total RNA and rRNA-depleted RNA were fragmented using RNAse III digestion for 13 min in a 10 μl reaction containing 1 μl of 10X RNAse III buffer and 20 U of RNAse III. After incubation the RNA was purified using the RiboMinus Concentration Module (Invitrogen), and the size and yield of RNA was determined using the Agilent bio-analyzer PICO chip. Library construction, emulsion PCR, workflow analysis and sequencing runs were performed following standard AB/Life Technologies protocols. A typical sequence run generated ~100 million reads of 50 nt each for long RNA and 30 nt long for short RNA, with the “strandedness” of the read on genomic DNA preserved.

### Read mapping

Sequenced reads were mapped onto the human genome assembly hg19 using the Burrows-Wheeler Alignment (BWA) algorithm [30]. Reads sequenced from the short RNA preparation were pre-processed using the *cutadapt* utility [76]. During mapping, all reads were quality-trimmed using each read’s associated quality values. Also, we allowed up to 2 mismatches in each read using BWA’s internal adaptive, read-length-dependent scheme. We did not allow any insertions or deletions. All reads that were mapped to the genome were post-processed, and those that landed on multiple locations (whether on the same or different chromosomes) were discarded and excluded from further consideration. The uniquely mapped reads can be examined at http://cm.jefferson.edu/platelets_2012/ by navigating to the genomic locus of interest; reads mapped to the forward strand are shown in blue, and reads mapped to the reverse strand in red.

### Estimating expression levels for transcripts

For each protein-coding transcript *T*, we determined the number of reads that uniquely mapped to *T*’s exons; distinct sequenced reads that mapped to the same genomic/exonic location of *T* were counted multiply. We defined the normalized expression (*ne*^*T*^*)* of *T by* the ratio (c^*T*^/*L*_*T*_)/(*c*^β-actin^/*L*_β*-actin*_), where c^*T*^ was the read count for *T*, *c*^β-actin^ was the read count for the β-actin transcript, and *L*_*T*_ and *L*_*β-actin*_ were the respective lengths of each transcript. For genes with multiple known protein-coding transcripts, the gene was assigned the *ne*^*T*^ value of its most abundant transcript. As described in the Results, we established that β-actin mRNA is abundantly and consistently expressed across samples. Throughout this study, we used the β-actin transcript with ENSEMBL identifier ENST00000331789 and the relationship of *ne*^*T*^ to that of *ne*^ENST00000331789^ to determine the presence or absence of protein-coding transcript *T* and, by extension, of the expression of the parent gene.

For non-coding transcripts, we used the same approach but instead of β-actin we used the levels of the small nucleolar RNA *SNORD44* as reference. This choice was informed by the abundance and apparent stability of *SNORD44*’s expression across many tissues and cell lines [77].

### Quantitative Reverse Transcription PCR (qRT-PCR) of Gene Expression

One microgram total RNA was reverse transcribed and 1% of the resulting cDNA (equivalent to 10 ng starting RNA) was used in the PCR. Quantitative reverse transcriptase PCR (qRT-PCR) results using primers specific for known platelet genes and for a panel of 89 genes encoding G-protein-coupled receptors are described in the Supplement. mRNA levels were assessed by the 2^-ΔΔCT^ method normalized to β-actin [78].

### Correlation between platelet RNA-seq and microarray datasets

The average log_2_-normalized expression of each long total RNA transcript across the 4 samples was ranked by transcript abundance and compared to published platelet transcript profiles obtained on Affymetrix GeneChip [32] and Illumina BeadChip microarray platforms [15,17]. A Spearman’s correlation coefficient was computed for the genes that are represented on all platforms.

### Enrichment analysis

To characterize the human platelet transcriptome with regard to possible over-representation of transcripts of a specific type, enrichment analysis was performed using the coordinates of those RNA-seq reads from both long and short total platelet RNA transcriptomes that could be mapped on the genome and the genomic coordinates of categories of transcripts as these are reported in the ENSEMBL database.

## Competing interests

The authors’ declare that they have no competing interests.

## Authors’ contributions

PFB, SEM, PF, and IR conceived of the study, and participated in its design and coordination. IR, PFB, PF, SEM, and LCE wrote the manuscript. LCE participated in the design and coordination, and acquired and analyzed the data. IR, H-WC, EL, PL, AE, and YJ acquired and analyzed the data. SN, KD, and JK participated in the design and acquired data. All authors read and approved the final manuscript.

## Supplementary Material

Additional file 1: Figure S1 Gene Ontology (GO) analysis of the platelet transcriptome from total RNA by RNASeq. **Figure S2.** The importance of distinguishing between the two products of a miRNA precursor. **Table S1**. Optimal choice of transcript for normalizing protein-coding expression. **Table S2.** Protein-coding transcripts in each of the four samples. Title and legend only **Table S3.** Impact of ribosomal RNA depletion on the estimate of mRNA abundance. Title and legend only. **Table S4.** MicroRNA transcripts in each of the four samples. Title and legened only. **Table S5.** Pseudogene expression in platelets. **Table S6.** Long platelet transcripts from repeat element regions. **Table S7.** Short platelet transcripts from repeat element regions. **Table S8.** Coordinates of protein-coding intronic regions that are not accounted for by the available annotations in the public databases. **Table S9.** Long platelet transcripts antisense to repeat element regions. **Table S10.** Mapping statistics for very lenient settings. Click here for file

Additional file 2: Table S2A Protein-coding transcripts in each of the four samples. List of the protein all coding transcripts whose expression is supported by the collected RNA-seq data. List for the four total RNA samples. In an effort to be conservative, and based on the data in Figures 1 and 2, we only considered protein-coding transcripts with an estimated abundance that was ≥ 2-10 times that of β-actin, and kept only those whose absolute ratio value was ≥ 2x between the two preparations. Click here for file

Additional file 3: Table S2B Protein-coding transcripts in each of the four samples. List of the protein all coding transcripts whose expression is supported by the collected RNA-seq data. List for the four rRNA-depleted samples. In an effort to be conservative, and based on the data in Figures 1 and 2, we only considered protein-coding transcripts with an estimated abundance that was ≥ 2-10 times that of β-actin, and kept only those whose absolute ratio value was ≥ 2x between the two preparations. Click here for file

Additional file 4: Table S3 Impact of ribosomal RNA depletion on the estimate of mRNA abundance. The table shows the base-2 logarithm of the ratio of the normalized expression between the total and rRNA-depleted preparation, and for each of the four individuals that provided the sequenced RNA. Only transcripts with an estimated abundance that is ≥ 2^-10^ times that of β-actin (ENST00000331789) and whose normalized expression differs by ≥ 2x (i.e. at least one log_2_ unit) between the preparations are listed. Click here for file

Additional file 5: Table S4 MicroRNA transcripts in each of the four samples. List of all microRNAs whose expression is supported by the collected RNA-seq data. Click here for file

Additional file 6 **Supplemental Materials.** Genomic coordinates and sequences of unannotated intronic regions that are found transcribed in human platelets. These are culled from both the long and short RNA-seq profiles. Click here for file
