# Supplementary material for: The complex transcriptional landscape of the anucleate human platelet
Source: BMC Genomics. 2013 Jan 16;14:1. doi: 10.1186/1471-2164-14-1 (PMC3722126; doi:10.1186/1471-2164-14-1)
Supplement: Additional file 1: Figure S1 — Gene Ontology (GO) analysis of the platelet transcriptome from total RNA by RNASeq. Figure S2. The importance of distinguishing between the two products of a miRNA precursor. Table S1. Optimal choice of transcript for normalizing protein-coding expression. Table S2. Protein-coding transcripts in each of the four samples. Title and legend only Table S3. Impact of ribosomal RNA depletion on the estimate of mRNA abundance. Title and legend only. Table S4. MicroRNA transcripts in each of the four samples. Title and legened only. Table S5. Pseudogene expression in platelets. Table S6. Long platelet transcripts from repeat element regions. Table S7. Short platelet transcripts from repeat element regions. Table S8. Coordinates of protein-coding intronic regions that are not accounted for by the available annotations in the public databases. Table S9. Long platelet transcripts antisense to repeat element regions. Table S10. Mapping statistics for very lenient settings. [file 1471-2164-14-1-S1.doc]

**SUPPLEMENTAL MATERIAL FOR:**

**The Complex Transcriptional Landscape of the Anucleate Human Platelet**

Paul F. Bray^1,*,✝^, Steven E. McKenzie^1,*^, Leonard Edelstein^1^, Srikanth Nagalla^1^, Kathleen Delgrosso^2^, Adam Ertel^2^, Joan Kupper^2^, Yi Jing^3^, Eric Londin^3^, Phillipe Loher^3^, Huang-Wen Chen^3^, Paolo Fortina^2#^, and Isidore Rigoutsos^3,#,✝^

^1^Cardeza Foundation for Hematologic Research, Division of Hematology, Department of Medicine, Thomas Jefferson University, Philadelphia, PA.

^2^Cancer Genomics Laboratory, Kimmel Cancer Center, Thomas Jefferson University, Philadelphia, PA.

^3^ Computational Medicine Center, Thomas Jefferson University, Philadelphia, PA.

^*^ These authors contributed equally to this work

^#^ These authors contributed equally to this work

^✝^ Correspondence: [paul.bray@jefferson.edu](mailto:paul.bray@jefferson.edu) or isidore.rigoutsos@jefferson.edu

**
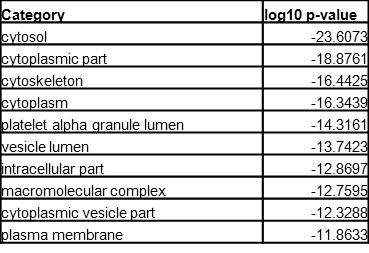

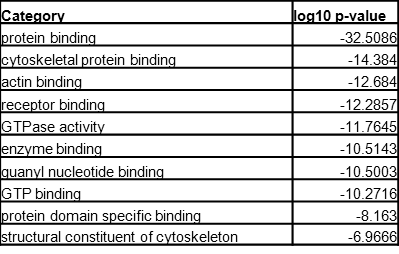

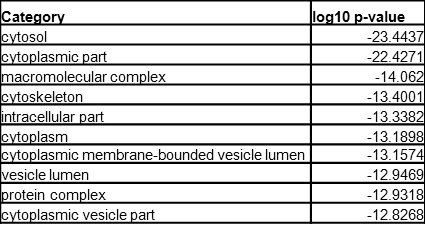

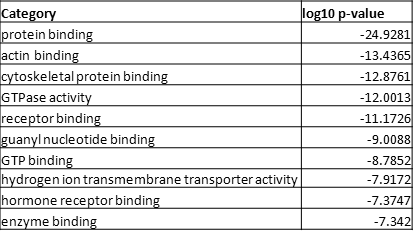
**

**B**

**# Genes in Category**

**0**

**500**

**1000**

**1500**

non-membrane-bounded organelle

membrane-enclosed lumen

cytosol

mitochondrion

organelle membrane

nucleoplasm

endoplasmic reticulum

cell fraction

Golgi apparatus

endomembrane system

**A**

**# Genes in Category**

**0**

**500**

**1000**

**1500**

**2000**

**2500**

ion binding

metal ion binding

transition metal ion binding

nucleotide binding

zinc ion binding

DNA binding

purine ribonucleotide binding

nucleoside binding

purine nucleoside binding

transcription regulator activity

**0**

**500**

**1000**

**1500**

non-membrane-bounded organelle

membrane-enclosed lumen

intracellular organelle lumen

cytosol

cytoskeleton

mitochondrion

organelle membrane

cell fraction

endoplasmic reticulum

Golgi apparatus

**# Genes in Category**

**0**

**500**

**1000**

**1500**

**2000**

**2500**

ion binding

metal ion binding

transition metal ion binding

nucleotide binding

zinc ion binding

DNA binding

purine ribonucleotide binding

nucleoside binding

purine nucleoside binding

transcription regulator activity

**# Genes in Category**

**D**

**C**

**F**

**E**

**H**

**G**

**Total RNA**

**rRNA-depleted RNA**

**Supplemental Figure S1. Gene Ontology (GO) analysis of the platelet transcriptome from total RNA by RNASeq.** GO analyses for Cellular Component (A, B, E, F) and Molecular Function (C, D, G, H), showing the numbers of genes in the most frequent gene categories (A, C, E, G) and the most enriched gene categories (B, D, F, H).

| 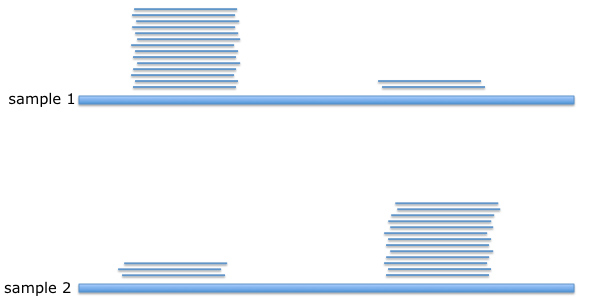 |
| --- |

**Supplemental Figure S2. The importance of distinguishing between the two products of a miRNA precursor.** RNA-seq gives us the ability to “see” that the two samples differ in the expression levels of the 5p and 3p products of this hypothetical miRNA precursor. But to do so, one needs to examine separately the genomic locations of each of the precursor’s two mature products (instead of treating the precursor as a single unit). Rationale is provided below.

**Rationale for Figure S2.** For many years, the primary, typically more abundant, miRNA product from a given miRNA precursor was referred to as the “mature miRNA”. The less abundant product from the precursor’s other arm was referred to as “miRNA*.” Over time, this led to significant confusion since the notation did not make apparent whether the mature miRNA originated from the left (5p) or right (3p) arm of the precursor. Beginning with the latest release of miRBase a new nomenclature was established: miRNA products from the left (resp. right) arm would be indicated by the suffix “-5p” (resp. “-3p”). Notation notwithstanding, the ability to distinguish between the two possible products of a microRNA precursor permits a more granular analysis of the sample under consideration. Surprisingly, what has been available in the public databases is a collection of the genomic coordinates for miRNA precursors, but for precursor product(s). Figure S2 highlights why using the *precursor*’s coordinates (instead of the precursor *product* coordinates) can lead to wrong conclusions. Shown are sequenced reads aligned to a hypothetical miRNA precursor. In sample 1, the majority of the reads come from the 5p arm of the precursor whereas in sample 2, the majority of the reads come from the 3p arm. If one treats the precursor as a unit, then the total number of reads remains unchanged between the two samples and thus no differential behavior will be reported at this genomic location. However, if we treat each of the two miRNA products separately in these samples we clearly arrive at a different conclusion. To facilitate our work in this regard, we have built and used in our analyses a tool that generates the genomic coordinates of each precursor product. We make the tool available for download from our website: <http://cm.jefferson.edu/downloadables/miRNACoord.tar.gz>.

**Supplemental Table S1. Optimal choice of transcript for normalizing protein-coding expression.** This table shows the base-2 logarithm of the ratio of the normalized expression between the total and rRNA-depleted preparation, and for each of the four individuals that provided the sequenced RNA. The calculations are reported for the protein-coding transcripts of both β-actin (*ACTB*) and the common normalizer *GAPDH*. Our chosen *ACTB* transcript (highlighted in blue) shows the least amount of variation.

| **Sample** | **transcript identifier** | **log_2_(ratio of normalized expression in**  **total vs. rRNA-depleted RNA)** |
| --- | --- | --- |
| 1 | ENSG00000075624_ENST00000331789_ACTB | 0.022368 |
| 2 | ENSG00000075624_ENST00000331789_ACTB | -0.043627 |
| 3 | ENSG00000075624_ENST00000331789_ACTB | 0.039259 |
| 4 | ENSG00000075624_ENST00000331789_ACTB | -0.000844 |
|  |  |  |
| 1 | ENSG00000075624_ENST00000320713_ACTB | 0.365838 |
| 2 | ENSG00000075624_ENST00000320713_ACTB | -0.096346 |
| 3 | ENSG00000075624_ENST00000320713_ACTB | 0.254189 |
| 4 | ENSG00000075624_ENST00000320713_ACTB | 0.128752 |
|  |  |  |
| 1 | ENSG00000075624_ENST00000400179_ACTB | 0.340914 |
| 2 | ENSG00000075624_ENST00000400179_ACTB | -0.107626 |
| 3 | ENSG00000075624_ENST00000400179_ACTB | 0.251881 |
| 4 | ENSG00000075624_ENST00000400179_ACTB | 0.121266 |
|  |  |  |
| 1 | ENSG00000075624_ENST00000432588_ACTB | -0.017631 |
| 2 | ENSG00000075624_ENST00000432588_ACTB | -0.564382 |
| 3 | ENSG00000075624_ENST00000432588_ACTB | 0.120984 |
| 4 | ENSG00000075624_ENST00000432588_ACTB | -0.043341 |
|  |  |  |
| 1 | ENSG00000075624_ENST00000445914_ACTB | 0.209814 |
| 2 | ENSG00000075624_ENST00000445914_ACTB | -0.214811 |
| 3 | ENSG00000075624_ENST00000445914_ACTB | 0.050579 |
| 4 | ENSG00000075624_ENST00000445914_ACTB | -0.028200 |
|  |  |  |
| 1 | ENSG00000111640_ENST00000229239_GAPDH | 0.398266 |
| 2 | ENSG00000111640_ENST00000229239_GAPDH | 0.718782 |
| 3 | ENSG00000111640_ENST00000229239_GAPDH | -0.771195 |
| 4 | ENSG00000111640_ENST00000229239_GAPDH | -0.428258 |
|  |  |  |
| 1 | ENSG00000111640_ENST00000396856_GAPDH | 0.472586 |
| 2 | ENSG00000111640_ENST00000396856_GAPDH | 0.732091 |
| 3 | ENSG00000111640_ENST00000396856_GAPDH | -0.744446 |
| 4 | ENSG00000111640_ENST00000396856_GAPDH | -0.396756 |
|  |  |  |
| 1 | ENSG00000111640_ENST00000396858_GAPDH | 0.588383 |
| 2 | ENSG00000111640_ENST00000396858_GAPDH | 1.210432 |
| 3 | ENSG00000111640_ENST00000396858_GAPDH | -0.682917 |
| 4 | ENSG00000111640_ENST00000396858_GAPDH | -0.345974 |
|  |  |  |
| 1 | ENSG00000111640_ENST00000396859_GAPDH | 0.468705 |
| 2 | ENSG00000111640_ENST00000396859_GAPDH | 0.852318 |
| 3 | ENSG00000111640_ENST00000396859_GAPDH | -0.712272 |
| 4 | ENSG00000111640_ENST00000396859_GAPDH | -0.365878 |
|  |  |  |
| 1 | ENSG00000111640_ENST00000396861_GAPDH | 0.404112 |
| 2 | ENSG00000111640_ENST00000396861_GAPDH | 0.718507 |
| 3 | ENSG00000111640_ENST00000396861_GAPDH | -0.750275 |
| 4 | ENSG00000111640_ENST00000396861_GAPDH | -0.393978 |

**Supplemental Table S2. Protein-coding transcripts in each of the four samples.** List of the protein all coding transcripts whose expression is supported by the collected RNA-seq data. 2A: list for the four total RNA samples. 2B: list for the four rRNA-depleted samples. In an effort to be conservative, and based on the data in Figures 1 and 2, we only considered protein-coding transcripts with an estimated abundance that was ≥ 2-10 times that of β-actin, and kept only those whose absolute ratio value was ≥ 2x between the two preparations.

(See separate spreadsheets: Suppl Table S2A, Total RNA, and

Suppl Table S2B, rRNA-depleted)

**Supplemental Table S3. Impact of ribosomal RNA depletion on the estimate of mRNA abundance.** The table shows the base-2 logarithm of the ratio of the normalized expression between the total and rRNA-depleted preparation, and for each of the four individuals that provided the sequenced RNA. Only transcripts with an estimated abundance that is ≥ 2^-10^ times that of β-actin (ENST00000331789) and whose normalized expression differs by ≥ 2x (i.e. at least one log_2_ unit) between the preparations are listed.

(See separate spreadsheet: Suppl Table S3)

**Supplemental Table S4. MicroRNA transcripts in each of the four samples.** List of all microRNAs whose expression is supported by the collected RNA-seq data.

(See separate spreadsheet: Suppl Table 4)

**Supplemental Table S5. Pseudogene expression in platelets.** Below we show the enrichments of the sequenced transcripts across the pseudogene loci using ENSEMBL63 annotations. Results are reported separately for the total and rRNA-depleted preparations. Note the consistently high enrichment across the four subjects and the two preparations.

|  |  | **span enrichment** |
| --- | --- | --- |
| **total RNA** | **sample 1** | 7.47 |
|  | **sample 2** | 10.97 |
|  | **sample 3** | 7.94 |
|  | **sample 4** | 3.13 |
| **rRNA depleted** | **sample 1** | 7.35 |
|  | **sample 2** | 10.16 |
|  | **sample 3** | 7.49 |
|  | **sample 4** | 2.92 |

**Supplemental Table S6. Long platelet transcripts from repeat element regions.** Below we show those of the repeat families that show evidence of sense transcription in the platelets’ *long* RNA-ome. Only families with enrichments ≥ 1.5 are shown. Note the consistency of the enriched families across the four subjects and the total and rRNA-depleted preparations.

|  | **repeat family** | **span enrichment** |
| --- | --- | --- |
| **sample 1 / Total RNA** | scRNA.scRNA | 62.02 |
|  | rRNA.rRNA | 45.23 |
|  | tRNA.tRNA | 16.12 |
|  | srpRNA.srpRNA | 15.93 |
|  | LINE.L1? | 5.25 |
|  | snRNA.snRNA | 4.51 |
|  | RNA.RNA | 4.33 |
|  | LTR?.LTR? | 2.76 |
|  | SINE?.SINE? | 1.62 |
|  |  |  |
| **sample 2 / Total RNA** | scRNA.scRNA | 244.44 |
|  | rRNA.rRNA | 219.32 |
|  | srpRNA.srpRNA | 67.02 |
|  | tRNA.tRNA | 32.75 |
|  | RNA.RNA | 13.20 |
|  | snRNA.snRNA | 5.05 |
|  | DNA.TcMar-Mariner | 2.36 |
|  | Simple_repeat.Simple_repeat | 1.66 |
|  | DNA.MuDR | 1.56 |
|  |  |  |
| **sample 3 / Total RNA** | rRNA.rRNA | 89.16 |
|  | scRNA.scRNA | 79.25 |
|  | srpRNA.srpRNA | 28.74 |
|  | tRNA.tRNA | 15.58 |
|  | RNA.RNA | 6.14 |
|  | snRNA.snRNA | 5.49 |
|  | DNA.Merlin | 1.72 |
|  | Unknown?.Unknown? | 1.66 |
|  |  |  |
| **sample 4 / Total RNA** | scRNA.scRNA | 18.07 |
|  | rRNA.rRNA | 14.82 |
|  | tRNA.tRNA | 7.20 |
|  | srpRNA.srpRNA | 5.35 |
|  | RNA.RNA | 3.83 |
|  | snRNA.snRNA | 3.43 |
|  | DNA.Merlin | 1.94 |
|  | LINE.L1? | 1.77 |
|  | SINE?.SINE? | 1.56 |

|  | **repeat family** | **span enrichment** |
| --- | --- | --- |
| **sample 1 / rRNA depleted** | scRNA.scRNA | 150.33 |
|  | rRNA.rRNA | 83.21 |
|  | tRNA.tRNA | 51.74 |
|  | srpRNA.srpRNA | 39.80 |
|  | snRNA.snRNA | 16.39 |
|  | RNA.RNA | 14.22 |
|  | LINE.L1? | 6.48 |
|  | Simple_repeat | 1.78 |
|  | Low_complexity | 1.55 |
|  |  |  |
| **sample 2 / rRNA depleted** | scRNA.scRNA | 213.13 |
|  | rRNA.rRNA | 80.81 |
|  | srpRNA.srpRNA | 55.52 |
|  | tRNA.tRNA | 39.90 |
|  | RNA.RNA | 11.27 |
|  | snRNA.snRNA | 10.33 |
|  | SINE?.SINE? | 1.87 |
|  | DNA.TcMar-Mariner | 1.70 |
|  |  |  |
| **sample 3 / rRNA depleted** | scRNA.scRNA | 78.64 |
|  | rRNA.rRNA | 52.49 |
|  | srpRNA.srpRNA | 30.48 |
|  | tRNA.tRNA | 17.51 |
|  | LINE.L1? | 8.22 |
|  | snRNA.snRNA | 7.37 |
|  | RNA.RNA | 6.51 |
|  |  |  |
| **sample 4 / rRNA depleted** | scRNA.scRNA | 14.28 |
|  | tRNA.tRNA | 7.78 |
|  | rRNA.rRNA | 7.31 |
|  | srpRNA.srpRNA | 5.59 |
|  | RNA.RNA | 3.86 |
|  | snRNA.snRNA | 2.96 |
|  | DNA.Merlin | 2.20 |
|  | SINE.tRNA | 1.67 |
|  | SINE?.SINE? | 1.66 |

**Supplemental Table S7.** **Short platelet transcripts from repeat element regions.** Below we show those of the repeat families that show evidence of sense transcription in the platelets’ *short* RNA-ome. Only families with enrichments ≥ 1.5 are shown. Note the consistency of the enriched families across the four subjects.

|  | **repeat family** | **span enrichment** |
| --- | --- | --- |
| **sample 1 / Short** | scRNA.scRNA | 46.15 |
|  | tRNA.tRNA | 26.53 |
|  | rRNA.rRNA | 13.88 |
|  | DNA.PiggyBac? | 3.01 |
|  | snRNA.snRNA | 2.95 |
|  | SINE?.SINE? | 2.28 |
|  | LTR?.LTR? | 2.12 |
|  | SINE.tRNA | 2.03 |
|  | srpRNA.srpRNA | 1.91 |
|  | LINE.L1? | 1.89 |
|  | LINE?.Penelope? | 1.86 |
|  | SINE.SINE | 1.64 |
|  | SINE.Deu | 1.62 |
|  | DNA.hAT-Blackjack | 1.60 |
|  | DNA.TcMar-Tc2 | 1.54 |
|  | DNA.hAT | 1.54 |
|  | DNA.TcMar-Mariner | 1.53 |
|  |  |  |
| **sample 2 / Short** | scRNA.scRNA | 57.88 |
|  | tRNA.tRNA | 36.17 |
|  | rRNA.rRNA | 17.92 |
|  | snRNA.snRNA | 3.15 |
|  | srpRNA.srpRNA | 2.95 |
|  | LTR?.LTR? | 2.79 |
|  | LINE?.Penelope? | 2.56 |
|  | Unknown?.Unknown? | 2.53 |
|  | DNA.PiggyBac? | 2.50 |
|  | LINE.RTE-BovB | 1.93 |
|  | DNA.Merlin | 1.91 |
|  | LINE.Dong-R4 | 1.89 |
|  | SINE.SINE | 1.86 |
|  | LTR.ERV | 1.83 |
|  | SINE.tRNA | 1.78 |
|  | DNA.hAT-Blackjack | 1.66 |
|  | LTR.ERVL? | 1.59 |
|  | RNA.RNA | 1.58 |
|  |  |  |
| **sample 3 / Short** | scRNA.scRNA | 43.16 |
|  | tRNA.tRNA | 26.74 |
|  | rRNA.rRNA | 14.52 |
|  | snRNA.snRNA | 3.23 |
|  | DNA.PiggyBac? | 2.84 |
|  | srpRNA.srpRNA | 2.20 |
|  | Unknown?.Unknown? | 2.07 |
|  | LINE?.Penelope? | 1.90 |
|  | SINE.SINE | 1.89 |
|  | LTR.ERV | 1.67 |
|  | RNA.RNA | 1.66 |
|  | SINE.tRNA | 1.62 |
|  | DNA.TcMar? | 1.57 |
|  | DNA.hAT-Blackjack | 1.52 |
|  |  |  |
| **sample 4 / Short** | scRNA.scRNA | 48.20 |
|  | tRNA.tRNA | 39.97 |
|  | rRNA.rRNA | 17.45 |
|  | snRNA.snRNA | 4.69 |
|  | Unknown?.Unknown? | 3.84 |
|  | LINE.L1? | 3.75 |
|  | srpRNA.srpRNA | 2.33 |
|  | LINE.RTE-BovB | 2.31 |
|  | DNA.PiggyBac? | 2.25 |
|  | LINE.Dong-R4 | 2.14 |
|  | RNA.RNA | 2.13 |
|  | SINE.tRNA | 1.99 |
|  | SINE?.SINE? | 1.94 |
|  | LTR?.LTR? | 1.83 |
|  | LTR.ERVL? | 1.68 |
|  | DNA?.DNA? | 1.62 |
|  | DNA.hAT-Blackjack | 1.62 |
|  | SINE.SINE | 1.58 |
|  | LTR.ERV | 1.55 |
|  | DNA.hAT? | 1.52 |
|  | DNA.TcMar? | 1.52 |
|  |  |  |

**Supplemental Table S8**. Coordinates of protein-coding intronic regions that are not accounted for by the available annotations in the public databases and are sources of abundant long and short transcripts.

DONE – TWO ASCII TABLES

(will be provided upon acceptance of manuscript)

**Supplemental Table S9. Long platelet transcripts antisense to repeat element regions.** Below we show those of the repeat element families that show evidence of *antisense* transcription in the platelets’ *long* RNA-ome. Only families with enrichments ≥ 1.5 are shown.

|  | **repeat family** | **span enrichment** |
| --- | --- | --- |
| **sample 1 / Total RNA** | DNA.Merlin | 3.23 |
|  | SINE.SINE | 2.31 |
|  |  |  |
| **sample 2 / Total RNA** | SINE?.SINE? | 2.51 |
|  | LINE.RTE-BovB | 1.56 |
|  | Simple_repeat.Simple_repeat | 1.53 |
|  |  |  |
| **sample 3 / Total RNA** | DNA.Merlin | 1.97 |
|  | SINE.SINE | 1.78 |
|  |  |  |
| **sample 4 / Total RNA** | DNA.TcMar | 1.89 |
|  | LINE.RTE-BovB | 1.77 |
|  | LINE.L1? | 1.65 |
|  |  |  |
| **sample 1 / rRNA depleted** | Simple_repeat.Simple_repeat | 1.88 |
|  | rRNA.rRNA | 1.75 |
|  | LINE.Dong-R4 | 1.65 |
|  | DNA.Merlin | 1.50 |
|  |  |  |
| **sample 2 / rRNA depleted** | rRNA.rRNA | 1.50 |
|  |  |  |
| **sample 3 / rRNA depleted** | DNA.Merlin | 3.39 |
|  | SINE.SINE | 1.62 |
|  |  |  |
| **sample 4 / rRNA depleted** | DNA.TcMar | 1.71 |
|  | LINE.L1? | 1.70 |
|  | LINE.RTE-BovB | 1.67 |

**Supplemental Table S10. Mapping statistics for very lenient settings.** Shown numbers are averages from platelet RNA for each library type and for all subjects. The “unmapped” reads correspond to those of the sequenced reads that, using standard parameter settings, cannot be mapped on the human genome either uniquely or multiply. See also main text.

| **Library** | **“Unmapped” reads** | **Uniquely mapped reads among the “unmapped”** |
| --- | --- | --- |
| Long, Total RNA | 43,673,084 | 4,932,663 (11.29%) |
| Long, rRNA-depleted RNA | 29,698,708 | 4,156,204 (13.99%) |
